# Intake of Different Dietary Proteins and Risk of Heart Failure in Men: The Kuopio Ischaemic Heart Disease Risk Factor Study

**DOI:** 10.1161/CIRCHEARTFAILURE.117.004531

**Published:** 2018-05-29

**Authors:** Heli E.K. Virtanen, Sari Voutilainen, Timo T. Koskinen, Jaakko Mursu, Tomi-Pekka Tuomainen, Jyrki K. Virtanen

**Affiliations:** Institute of Public Health and Clinical Nutrition, University of Eastern Finland, Kuopio.

**Keywords:** calcium, dietary proteins, heart failure, men, prospective studies

## Abstract

**Background:**

Animal and plant protein intakes have indicated opposite associations with cardiovascular mortality risk. Whether dietary proteins are associated with risk of heart failure (HF) is unclear. Thus, we examined the associations of proteins from different food sources with risk of HF.

**Methods and Results:**

The study included 2441 men aged 42 to 60 years at the baseline examinations in 1984 to 1989 in the Kuopio Ischaemic Heart Disease Risk Factor Study. Protein intakes at baseline were assessed with 4-day dietary records. Data on incident HF cases were obtained from national registers. HF risk according to protein intake was estimated by Cox proportional hazard ratios. During the mean follow-up of 22.2 years, 334 incident HF cases occurred. Higher intake of total protein indicated a trend toward increased risk of HF (multivariable-adjusted hazard ratio in the highest versus lowest quartile=1.33; 95% confidence interval: 0.95–1.85; *P*-trend=0.05). The associations between specific types and sources of protein with incident HF were consistent with this overall finding although not all associations reached statistical significance. For example, the hazard ratio in the highest versus lowest quartile was 1.43 (95% confidence interval: 1.00–2.03; *P*-trend=0.07) for total animal protein and 1.17 (95% confidence interval: 0.72–1.91; *P*-trend=0.35) for total plant protein.

**Conclusions:**

In middle-aged men, higher protein intake was marginally associated with increased risk of HF.

**CLINICAL TRIAL REGISTRATION:**

URL: https://www.clinicaltrials.gov. Unique identifier: NCT03221127

## Abstract 2

Supplemental Digital Content is available in the text.

**WHAT IS NEW?**

- Despite the increasing popularity of high-protein diets, the role of dietary protein in relation to heart failure risk has not been previously evaluated.
- We observed that high total protein intake was marginally associated with increased risk of heart failure.
- The associations with proteins from different sources were in accordance with the overall finding, although not all associations reached statistical significance.

**WHAT ARE THE CLINICAL IMPLICATIONS?**

- This study suggests that high protein intake may not be the optimal dietary strategy in the prevention of heart failure.

Despite the achieved improvements in heart failure (HF) management, the disease dramatically shortens life expectancy,^1^ highlighting the need for efficient prevention. Investigations on dietary approaches concentrating on HF prevention are scarce. Prospective studies have suggested that greater intake of fish or marine omega-3 polyunsaturated fatty acids^2,3^ and whole grain products^4,5^ may associate with lower HF risk. In contrast, greater intake of total^2^ or red meat^6^ and especially processed red meat^7,8^ or frequent intake of eggs^9^ may associate with increased HF risk. Results from previous studies are, nevertheless, inconclusive, and it is unclear which factors explain the differential relations of the protein sources with HF risk.

One factor contributing to cardiac function and onset of HF may be dietary protein. Substituting either plant or animal protein for carbohydrates has lowered blood pressure in experimental studies.^10,11^ Because high blood pressure is a major risk factor for HF,^1^ protein intake could also play a beneficial role in the pathogenesis of HF. Benefits are suggested also by other studies: sufficient protein intake (>0.7 g/kg ideal body weight) was associated with lower cardiovascular and all-cause mortality in hypertensive patients,^12^ and amino acid supplementation prevented cardiac dysfunction in patients with type 2 diabetes mellitus.^13,14^

Instead of considering only the total protein intake, prospective studies have indicated that proteins from different dietary sources may be differentially related to health. For example, higher intake of animal protein has been associated with increased cardiovascular mortality in some observational studies^15,16^ while plant protein intake has had an inverse association.^15,17^ Whether different proteins with specific amino acid compositions have distinct roles also in relation to HF risk remains to be established.

To elucidate the role of dietary proteins in risk of HF, we examined whether total, animal, or plant protein intakes are related to HF risk in a population of middle-aged and older Finnish men. We also compared the associations of proteins from more specific animal and plant sources. In the secondary analyses, we investigated the associations of intakes of the main dietary protein sources with HF risk.

## Methods

The data, analytic methods, and study materials will not be made available to other researchers for purposes of reproducing the results or replicating the procedure.

### Study Population

The KIHD (Kuopio Ischaemic Heart Disease Risk Factor Study) was designed to investigate risk factors for cardiovascular disease (CVD), atherosclerosis, and related outcomes in a population based, randomly selected sample of men from eastern Finland.^18^ The baseline examinations were performed in 1984 to 1989. A total of 2682 men who were 42, 48, 54, or 60 years old at baseline (83% of those eligible) were recruited in 2 cohorts (Figure I in the Data Supplement). Re-examinations were conducted 4, 11, and 20 years after the baseline. The baseline characteristics of the entire study population have been described previously.^18^ The Research Ethics Committee of the University of Kuopio has approved the KIHD study, and dietary compound of KIHD has been registered at Clinicaltrials.gov. All subjects gave written informed consent. Subjects with diagnosed HF at baseline (n=194), unknown HF status (n=7), and those with missing data on dietary intakes (n=40) were excluded, leaving 2441 men for analyses.

### Baseline Measurements

Fasting venous blood samples were collected between 8 am and 10 am. Subjects were instructed to abstain from ingesting alcohol for 3 days and from smoking and eating for 12 hours before providing the sample. Detailed descriptions of the determination of serum lipids,^19^ lipoproteins,^19^ and magnesium^20^ and the assessment of medical history and medications,^19^ family history of diseases,^19^ smoking,^19^ alcohol consumption,^19^ physical activity,^21^ and blood pressure^19^ have been published. Education years, annual income, marital status, and dietary supplement use were obtained from self-administered questionnaires. Family history of coronary heart disease (CHD) was defined as positive if a first-degree relative of the participant had a CHD history. Body mass index (BMI) was computed as the ratio of weight in kilograms to the square of height in meters. Serum creatinine was measured with the clinical chemistry analyzer Kone Specific (KONE Instruments Oy, Espoo, Finland) using Jaffe reaction, and estimated glomerular filtration rate was calculated by the Chronic Kidney Disease Epidemiology Collaboration formula.^22^

### Dietary Assessment

Baseline food consumption was assessed with a food record of 4 days, one of which was a weekend day, by using household measures. A picture book of common foods and dishes was used to help in the estimation of portion sizes. To further improve accuracy, instructions were given, and completed records were checked by a nutritionist together with the participant. Nutrient intakes were estimated with NUTRICA 2.5 software (Social Insurance Institution, Turku, Finland). The software’s databank is mainly based on Finnish nutrient composition values of foods.

We calculated protein coming from different sources (Table I in the Data Supplement). Total meat included red meat, white meat, and offal. Processed red meat included industrially processed red meat. For the analyses with major plant protein sources, we combined the most protein-rich plant foods, that is, grain products, legumes, nuts, and seeds. Dietary calcium included calcium from food sources but not from supplements. Each nutrient was energy adjusted by the residual method.^23^

### Ascertainment of HF Cases and Other Diseases

Incident HF cases between study entry and December 31, 2014, were obtained by computer linkage to the National Hospital Discharge Register (maintained by the National Institute for Health and Welfare in Finland). The *International Classification of Disease*, *Tenth Revision* codes I11.0 and I50.0-I50.9 were used for diagnostic classification of HF cases. There were no losses to follow-up.

Data on other incident diseases or medications during the follow-up were obtained by record linkage to the National Hospital Discharge Register and to the Social Insurance Institution of Finland register for reimbursement of medicine expenses or by diagnosis at the re-examination rounds.

### Statistical Analysis

The univariate relations of total, animal, and plant protein intakes with baseline characteristics were assessed by means and linear regression (continuous, normally distributed variables), by medians and Jonckheere–Terpstra test (continuous, skewed variables), or by χ^2^ tests (categorical variables). Correlations between intakes of different proteins were estimated by Spearman correlation coefficients.

Person-years of follow-up were calculated from the baseline to the date of HF diagnosis, death, or the end of follow-up, whichever came first. Cox proportional hazards regression models were used to estimate hazard ratios (HRs) in exposure quartiles, with the lowest category as the reference. Schoenfeld residuals did not indicate significant evidence of violation of the proportional hazards assumption. Absolute risk (AR) change was calculated by multiplying the AR in the reference group by the multivariable-adjusted HR change in the comparison group.

Covariate selection was based on literature of identified and potential risk factors for HF or on associations with exposures and outcomes in the present analysis. Model 1 included age, examination year, and energy intake. The multivariable model (model 2) included the variables in model 1 plus education years, income, pack-years of smoking, alcohol intake, leisure-time physical activity, BMI, family history of CHD, baseline disease status (CHD or use of cardiac medications, diabetes mellitus or hypertension), and intakes of saturated, monounsaturated, polyunsaturated, and trans fatty acids and fiber. In addition, the disease status was updated throughout the follow-up, and the diseases and medications mentioned above were included as time-dependent covariates. Model 2 was also mutually adjusted for other proteins. Models that include protein and fat but not carbohydrates can be interpreted as replacement of carbohydrates with the protein in interest. All quantitative variables were entered in the models as continuous variables. The cohort mean was used to replace missing values in covariates (<2.4%). Tests of linear trend were conducted by assigning the median values for each category of exposure variable and treating those as a single continuous variable. Marital status, use of dietary supplements, serum total cholesterol/high-density lipoprotein cholesterol ratio, serum triglycerides, serum magnesium, baseline estimated glomerular filtration rate, history of atrial fibrillation, cardiomyopathy, stroke, valvular defect, myocarditis or chronic obstructive pulmonary disease, or intake of fruits and vegetables were not included in the models because they did not appreciably affect the associations (change in estimates <5%). Potential nonlinear associations were assessed semiparametrically using restricted cubic splines with 3 knots. To assess replacement of proteins with each other, all proteins were simultaneously added into the models, and the difference of regression coefficients of 2 proteins of interest, their variance, and covariance were used for calculating HRs and 95% confidence intervals (CIs).

In the secondary analyses, we investigated the associations of the protein sources with risk of HF. Identical list of covariates were used as in the main analysis. However, the possible effect mediators, that is, intakes of saturated, monounsaturated, polyunsaturated, and trans fatty acids and fiber, were not added to model 2, but instead an additional model 3 with these factors was created.

The statistical significance of the interactions with baseline disease status (CHD or use of cardiac medications, diabetes mellitus, or hypertension), CHD status, BMI (below versus above median), and smoking status was assessed by likelihood ratio tests with the use of a cross-product term. All *P* values were 2-tailed (α=0.05). Data were analyzed with SPSS 21.0 for Windows (IBM Corp, Armonk, NY) and Stata 13.1 (Stata Corp, College Station, TX; for spline analysis).

## Results

### Baseline Characteristics

The mean protein intake was 93.2 g/d (15.8% of total energy intake), of which 70.0% was from animal sources and 27.7% from plant sources (Table II in the Data Supplement). Of the total protein intake, 2.3% was from mixed sources and was not included into either animal or plant protein. Dairy (28.8 g/d), meat (24.7 g/d), and fish (8.1 g/d) were the major animal protein sources whereas most of the plant protein came from grain products (20.5 g/d) and potatoes (2.4 g/d).

Table 1 shows the baseline characteristics of the study population. Men with greater total protein intake were younger, more likely to be married, had longer education, and higher income than those with lower protein intake. On the contrary, they had higher BMI and were more likely to have diabetes mellitus. They had higher intake of fiber, polyunsaturated fatty acids, fruits, berries and vegetables, and processed red meat. High animal protein intake was also associated with more favorable socioeconomic factors, but with higher BMI, higher probability of being smoker and having diabetes mellitus. Those with higher animal protein intake had lower intake of fiber but higher intake of polyunsaturated fatty acids. High plant protein intake was generally associated with healthier lifestyle and dietary factors (Table 1).

**Table 1.**
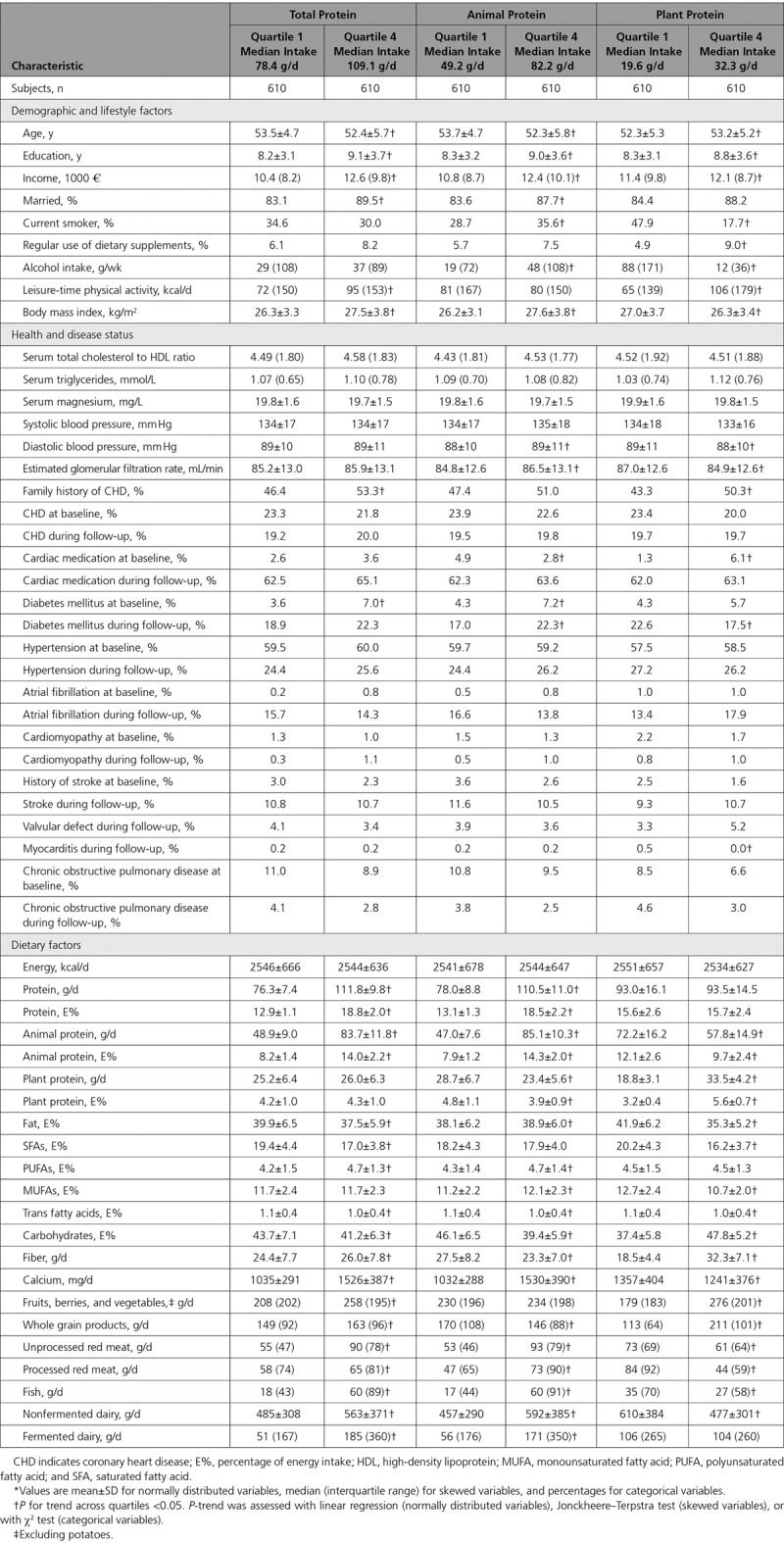
Baseline Characteristics According to Total, Animal, and Plant Protein Intake Among 2441 Men From the Kuopio Ischemic Heart Disease Risk Factor Study*

### Associations of Dietary Proteins With Risk of HF

During the mean follow-up of 22.2 years, 334 cases of HF were recorded. Total protein intake was not associated with risk of HF in the age, examination year, and energy intake adjusted model (model 1; Table 2). After multivariable adjustments, those in the highest versus the lowest quartile of total protein had a borderline significant 33% (95% CI: −5% to 85%; *P*-trend=0.05) increased risk of HF. There was no evidence for nonlinearity in the association (Figure). Those in the highest versus the lowest quartile of total animal protein had 43% increased risk (95% CI: 0%–103%; *P*-trend=0.07); AR=12.3% in the lowest quartile, with a 5.3% increase in the highest quartile to AR=17.6%; model 2 in Table 2.

**Table 2.**
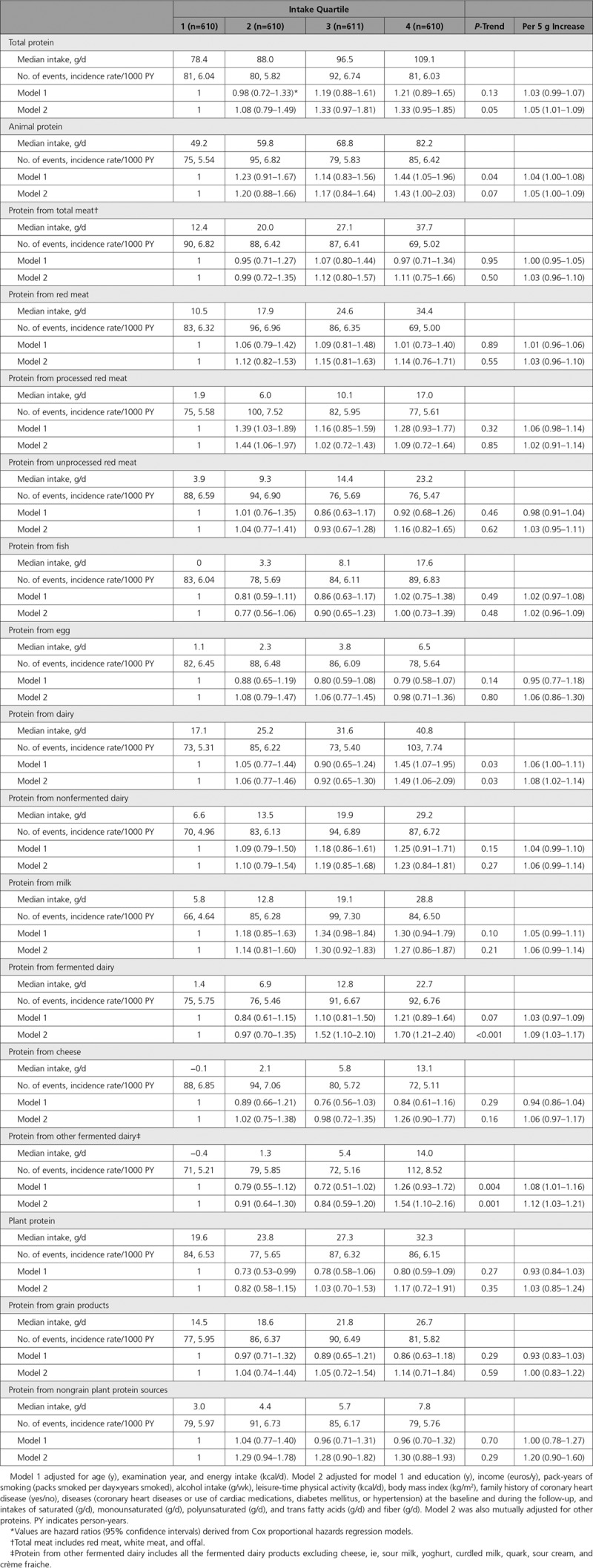
Risk of Incident Heart Failure According to Protein Intake Among 2441 Men From the Kuopio Ischemic Heart Disease Risk Factor Study

**Figure.**
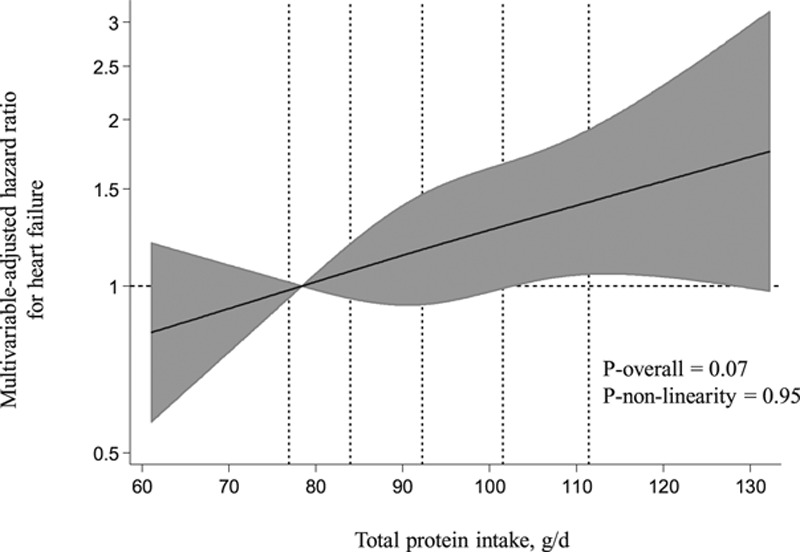
**Multivariable-adjusted hazard ratios of total protein intake with risk of heart failure in 2441 men, evaluated by restricted cubic splines from Cox proportional hazards models.** The model was adjusted for age (y), examination year, energy intake (kcal/d), education (y), income (euros/y), pack-years of smoking (packs smoked per day×years smoked), alcohol intake (g/wk), leisure-time physical activity (kcal/d), body mass index (kg/m^2^), family history of coronary heart disease (yes/no), diseases (coronary heart diseases or use of cardiac medications, diabetes mellitus, or hypertension) at the baseline and during the follow-up, and intakes of saturated (g/d), monounsaturated (g/d), polyunsaturated (g/d), and trans fatty acids (g/d) and fiber (g/d). The solid lines represent the central risk estimates, and the shades are the 95% confidence interval, relative to the reference level (12.5th percentile). The dotted vertical lines correspond to 10th, 25th, 50th, 75th, and 90th percentile of the total protein intake.

Those in the highest versus lowest quartile of dairy protein intake had multivariable-adjusted 49% (6%–109%; *P*-trend=0.03) higher risk of HF (model 2 in Table 2; AR=12.0% in the lowest quartile, AR=17.9% in the highest quartile). However, there was evidence for nonlinearity (*P*-nonlinearity=0.03; Figure II in the Data Supplement). The association was stronger for protein from fermented dairy, with 70% (95% CI: 21%–140%; *P*-trend <0.001; *P*-nonlinearity=0.09) higher risk in the highest quartile as protein from nonfermented dairy indicated nonsignificant association (Table 2). Because of these findings, we explored the baseline characteristics according to the fermented dairy protein intake but found that higher intake of fermented dairy protein was generally associated with healthier lifestyle and dietary factors (Table III in the Data Supplement). In more detailed analyses, protein from other fermented dairy products (98.4% contained ≤2.5% fat) had a stronger association with HF than protein from cheese (Table 2). Proteins from total meat and from meat subtypes, milk, and plant sources had nonsignificant associations toward increased HF risk (Table 2), without evidence for nonlinear associations (*P*-nonlinearity >0.10). Proteins from fish and eggs were not associated with HF risk.

In the substitution models, replacing protein from one source with protein from another source did not reveal any statistically significant associations (*P*>0.05).

### Associations of Dietary Protein Sources With Risk of HF

Intake of total dairy had a trend toward increased risk of HF in multivariable-adjusted model 2, with a 36% (95% CI: −5% to 94%; *P*-trend=0.07) higher risk in the highest versus the lowest quartile. Adjusting for nutrient intakes strengthened the association (model 3; Table IV in the Data Supplement). Those in the highest intake quartile of fermented dairy products had multivariable-adjusted 62% (95% CI: 20%–119%; *P*-trend=0.001) higher risk of HF when compared with the lowest quartile (model 2; Table IV in the Data Supplement). Further adjustment for nutrients had little impact on the associations (model 3). Nonfermented dairy intake had no association with HF risk (Table IV in the Data Supplement). Intakes of total meat or any meat subtype, fish, eggs, or main vegetable protein sources did not have statistically significant associations with HF risk in the multivariable-adjusted models (Table IV in the Data Supplement).

### Association of Calcium Intake With Risk of HF

Because of the findings with dairy, in the post hoc analyses, we investigated the associations of dietary calcium with HF risk. After multivariable adjustments, those in the highest calcium intake quartile had 39% (95% CI: 1%–91%; *P*-trend=0.07) higher risk of HF when compared with the lowest quartile (model 2; Table V in the Data Supplement). The increased risk was only present in the highest calcium intake quartile (>1528 mg/d).

We also tested whether adjustment for calcium intake attenuates the associations observed with fermented dairy protein. Adjusting model 2 further for calcium intake, the HRs (95% CIs) in the highest quartile for proteins from fermented dairy and fermented dairy excluding cheese were 1.86 (1.00, 3.55) and 1.46 (0.87, 2.47), respectively.

### Sensitivity Analyses

Dairy, fermented dairy, or any dairy proteins did not indicate statistically significant interactions by baseline disease history (CHD or use of cardiac medications, diabetes mellitus or hypertension; *P*-interactions >0.32), CHD history (*P*-interactions >0.32), BMI (*P*-interactions >0.47), or smoking status (*P*-interactions >0.62). However, in the stratified analyses based on disease history, higher intake of the major plant protein sources was associated with increased risk of HF among those without disease history (n=832; 64 cases; HR per 100 g intake, 1.46; 95% CI: 1.00–2.12; model 2) but not among those with disease history (n=1609; 270 cases; HR=0.91; 95% CI: 0.76–1.10; *P*-interaction=0.04). We did not find evidence for interaction with intake of plant protein (*P*-interaction=0.13). For other proteins or protein sources, interactions by disease history (*P*-interactions >0.07), CHD history (*P*-interactions >0.08), BMI (*P*-interactions >0.06), or smoking status (*P*-interactions >0.15) were not statistically significant.

Because HF is typically an end-stage heart disease, some of the CVD subjects may die before developing HF. Thus, we considered the competing risk of CVD mortality by using a composite outcome of HF or CVD mortality (n=1640; cases=379 after excluding the participants with CVD at baseline). In these analyses, most associations were attenuated (eg, HR=1.04; 95% CI: 1.00–1.08 per 5 g higher total protein intake) whereas the association for nonfermented dairy was strengthened (Table VI in the Data Supplement).

Because the long follow-up may attenuate associations with the exposures that were assessed only at baseline, we tested the associations with 10 years shorter follow-up (n=96 cases). The associations were generally similar although not all of them reached statistical significance. For example, HRs (95% CIs) per 5 g intake of total, animal, dairy, and fermented dairy protein were 1.06 (0.98–1.15), 1.06 (0.97, 1.14), 1.10 (0.99, 1.22), and 1.13 (1.01, 1.27), respectively (model 2; other data not shown). The association of cheese intake with risk of HF was stronger with the shorter follow-up (model 2 HR=1.58; 95% CI: 1.03–2.41 per 50 g intake of cheese), and the association remained statistically significant after adjustment for nutrient intakes. We also tested the exclusion of HF cases that occurred during the first 2 years of follow-up (n=3) but this did not change the results.

## Discussion

In this population-based cohort study in middle-aged and older men from eastern Finland, high protein intake was marginally associated with increased risk of HF. Most proteins from different animal and plant sources were associated with a higher risk although not all associations were statistically significant. Similar findings were observed with the protein sources.

Previously, high-protein–low-carbohydrate diets have been related to increased risk of type 2 diabetes mellitus and all-cause mortality,^17^ and high animal protein intake with increased cardiovascular mortality,^15,16^ but studies have not examined protein intake in relation to HF risk. Nevertheless, trials in humans have suggested that supplementation with a mixture of amino acids may prevent cardiac dysfunction in patients with type 2 diabetes mellitus.^13,14^ Of various amino acids, branched chain amino acids (BCAAs) that are abundant in dairy but also in other animal protein sources^24^ are of special interest as BCAA catabolism is impaired in a failing heart.^25,26^ The unanswered question is whether the dietary intake of BCAAs affects the development of HF.^26^ Human trials are lacking, and BCAA supplementation in animals has had both beneficial^27,28^ and harmful effects on cardiac function.^29^ Thus, the potential effects of proteins and amino acids on HF risk need further clarification.

In our analyses, total dairy and fermented dairy were associated with increased HF risk. As we observed comparable associations for dairy and dairy proteins, it is hard to disentangle whether the results are because of the protein per se or because of some other factors in dairy. In one previous study, intake of high-fat dairy was related to increased HF risk.^5^ However, total^2^ or fermented dairy^30,31^ intake has not been related to HF risk in other studies. Furthermore, studies have reported beneficial or neutral associations of dairy and fermented dairy intakes with risk of type 2 diabetes mellitus and CVD.^32–34^ Also we have observed in this same cohort that high intake of fermented dairy was associated with decreased risk of fatal and nonfatal CHD.^35^ Dairy protein and major dairy amino acids have also induced beneficial metabolic effects in trials, such as lowered blood pressure and improved glycemic control.^11,36–38^ Interestingly, though a meta-analysis observed an increased risk of all-cause mortality with high dairy intakes (>750 mL/d),^39^ indicating that high intakes may not be favorable. Accordingly, we observed higher HF risk mainly among those with the highest intake of total (>927 g/d) and fermented dairy (>281 g/d). It is worth noticing that the median intakes of total dairy (682 g/d) and fermented dairy (103 g/d) in our study are higher than in many other cohorts, where the corresponding intakes have been 111 to 400 g/d and 40 to 100 g/d, respectively.^34^

What could explain the stronger association of fermented compared with nonfermented dairy with HF risk in our study? A major source of fermented dairy was sour milk whereas nonfermented dairy was predominantly milk. These products have fairly similar nutrient content (eg, BCAAs, other amino acids, calcium), indicating that these nutrients unlikely account for the stronger association of fermented dairy. Moreover, as adjustment for different dietary fatty acids strengthened the direct association between fermented dairy and risk of HF, the fat quality is not a probable explanation, either, especially because the association was stronger with protein coming predominantly from reduced or low-fat fermented dairy products. Sour milk also includes probiotics, but probiotics may—based on animal studies—actually have beneficial effects on cardiac function,^40^ thus providing no clarification for our observation. Finally, as higher fermented dairy protein intake was associated with a healthier lifestyle, other lifestyle factors unlikely explain its association with higher HF risk. To sum up, we did not find a plausible explanation for why fermented dairy or its protein would be more detrimental than nonfermented dairy. Thus, considering that we included many analyses in the present study, we cannot exclude the possibility of a chance finding explaining the result.

In the post hoc analyses, a high intake of dietary calcium (>1528 mg/d) was related to increased HF risk. In contrast, calcium supplementation (1000 mg/d) with vitamin D (400 µg/d) did not affect HF risk in the overall cohort of postmenopausal women but was beneficial for those with low HF risk.^41^ However, a meta-analysis observed a U-shaped association between total calcium intake and cardiovascular mortality: the risk gradually increased at intakes >800 mg/d.^42^ Studies, however, suggest that it is mainly the excessive intake of supplemental calcium that associates with increased cardiovascular risk whereas dietary calcium is generally considered safe.^43–45^ The more pronounced increase in serum calcium after calcium supplementation is hypothesized to promote aortic calcification and blood coagulation.^44^ Because we did not have data about calcium supplement use in our cohort, we were not able to compare dietary and supplemental calcium. However, higher dietary calcium intake compared with most studies^42^ may possibly explain its association with increased HF risk.

In agreement with one previous meta-analysis,^46^ we did not observe protein from fish or fish intake to associate with HF risk. In contrast, another meta-analysis found that intake of fish and marine omega-3 fatty acids is related to decreased HF risk.^3^ Preparation methods could explain the discrepancy in results; baked or broiled fish has been associated with decreased HF risk whereas fried fish has had an association with increased risk.^47,48^ Intakes of meat protein, total meat, or any meat subtype were not associated with HF risk, either. This result complies with one previous study, in which intake of red meat was not associated with HF risk.^5^ Yet, many studies have found a higher HF risk with greater intakes of total meat,^2^ red meat,^6^ and especially of processed red meat.^7,8^ Because consumption of total and processed meat in our study is comparable or higher than in other cohorts,^2,8^ the meat consumption patterns unlikely explain the difference between the studies. Differences in preparation methods of meat and difficulties in categorizing meat as unprocessed or processed^49^ may, however, affect the results. There is no apparent explanation for why a high consumption of major plant sources was associated with a higher HF risk among those without disease history. Because of low number of events in this group, the association might be a chance finding.

Our finding that egg protein or egg intake had no association with HF risk contradicts with a meta-analysis that revealed a direct association between high egg intake and HF risk.^9^ Egg contains high quality protein and several bioactive compounds but is also a major source of dietary cholesterol and hence still has a controversial role on heart health and mortality.^39,50^ Lifestyle factors related to egg intake may also affect its associations with health.^50^ More research on effects of egg intake in diverse populations is thus needed.^50^

The strengths of our study are the prospective population-based setting, no losses during the follow-up, and extensive measurement of diet and possible confounding factors. Our study also has limitations. The single baseline measurement of diet and other lifestyle factors and the inability of a 4-day food recording to accurately assess typical intakes of occasionally consumed and seasonally varying foods could introduce random error and thus attenuate associations. However, the associations were comparable with a shorter follow-up. Despite considering various covariates in our models, residual confounding cannot be completely excluded, and it may potentially explain the associations between dairy and HF. We also did not have information on calcium supplement use. Some misclassification in the outcome measure is possible although the use of national registers for ascertaining HF cases reduces false diagnoses compared with self-reporting. Finally, we were not able to separate the HF cases with preserved or reduced ejection fraction that have differential pathogenesis^1^ and cannot, therefore, conclude whether the associations of protein intake differ with different subtypes of HF.

## Conclusions

Our results suggest that higher protein intake may be associated with a higher risk of HF in middle-aged and older men. Further studies in diverse study populations are needed to elucidate the role of protein intake in the pathogenesis of HF.

## Acknowledgments

We thank Ari Voutilainen, PhD, for assistance with statistical analysis.

## Sources of Funding

This study was supported by Finnish Cultural Foundation North Savo Regional fund (H.E.K. Virtanen), Päivikki and Sakari Sohlberg Foundation (H.E.K. Virtanen), Paavo Nurmi Foundation (H.E.K. Virtanen), and the Finnish Association of Academic Agronomists (H.E.K. Virtanen).

## Disclosures

None.

## Supplementary Material

## References

[1] Bui AL, Horwich TB, Fonarow GC (2011). Epidemiology and risk profile of heart failure.. Nat Rev Cardiol.

[2] Wirth J, di Giuseppe R, Boeing H, Weikert C (2016). A Mediterranean-style diet, its components and the risk of heart failure: a prospective population-based study in a non-Mediterranean country.. Eur J Clin Nutr.

[3] Djoussé L, Akinkuolie AO, Wu JH, Ding EL, Gaziano JM (2012). Fish consumption, omega-3 fatty acids and risk of heart failure: a meta-analysis.. Clin Nutr.

[4] Djoussé L, Gaziano JM (2007). Breakfast cereals and risk of heart failure in the physicians’ health study I.. Arch Intern Med.

[5] Nettleton JA, Steffen LM, Loehr LR, Rosamond WD, Folsom AR (2008). Incident heart failure is associated with lower whole-grain intake and greater high-fat dairy and egg intake in the Atherosclerosis Risk in Communities (ARIC) study.. J Am Diet Assoc.

[6] Ashaye A, Gaziano J, Djoussé L (2011). Red meat consumption and risk of heart failure in male physicians.. Nutr Metab Cardiovasc Dis.

[7] Kaluza J, Åkesson A, Wolk A (2015). Long-term processed and unprocessed red meat consumption and risk of heart failure: a prospective cohort study of women.. Int J Cardiol.

[8] Kaluza J, Akesson A, Wolk A (2014). Processed and unprocessed red meat consumption and risk of heart failure: prospective study of men.. Circ Heart Fail.

[9] Khawaja O, Singh H, Luni F, Kabour A, Ali SS, Taleb M, Ahmed H, Gaziano JM, Djoussé L (2017). Egg consumption and incidence of heart failure: a meta-analysis of prospective cohort studies.. Front Nutr.

[10] Tielemans SM, Altorf-van der Kuil W, Engberink MF, Brink EJ, van Baak MA, Bakker SJ, Geleijnse JM (2013). Intake of total protein, plant protein and animal protein in relation to blood pressure: a meta-analysis of observational and intervention studies.. J Hum Hypertens.

[11] Fekete ÁA, Giromini C, Chatzidiakou Y, Givens DI, Lovegrove JA (2016). Whey protein lowers blood pressure and improves endothelial function and lipid biomarkers in adults with prehypertension and mild hypertension: results from the chronic Whey2Go randomized controlled trial.. Am J Clin Nutr.

[12] Courand PY, Lesiuk C, Milon H, Defforges A, Fouque D, Harbaoui B, Lantelme P (2016). Association between protein intake and mortality in hypertensive patients without chronic kidney disease in the OLD-HTA cohort.. Hypertension.

[13] Scognamiglio R, Negut C, Piccolotto R, Dioguardi FS, Tiengo A, Avogaro A (2004). Effects of oral amino acid supplementation on myocardial function in patients with type 2 diabetes mellitus.. Am Heart J.

[14] Scognamiglio R, Negut C, Palisi M, Dioguardi FS, Coccato M, Iliceto S (2008). Effects of oral amino acid supplements on cardiac function and remodeling in patients with type 2 diabetes with mild-to-moderate left ventricular dysfunction.. Am J Cardiol.

[15] Song M, Fung TT, Hu FB, Willett WC, Longo VD, Chan AT, Giovannucci EL (2016). Association of animal and plant protein intake with all- cause and cause-specific mortality.. JAMA Intern Med.

[16] Hernández-Alonso P, Salas-Salvadó J, Ruiz-Canela M, Corella D, Estruch R, Fitó M, Arós F, Gómez-Gracia E, Fiol M, Lapetra J, Basora J, Serra-Majem L, Muñoz MÁ, Buil-Cosiales P, Saiz C, Bulló M (2016). High dietary protein intake is associated with an increased body weight and total death risk.. Clin Nutr.

[17] Pedersen AN, Kondrup J, Borsheim E (2013). Health effects of protein intake in healthy adults: a systematic literature review.. Food Nutr Res.

[18] Salonen JT (1988). Is there a continuing need for longitudinal epidemiologic research? The Kuopio Ischaemic Heart Disease Risk Factor Study.. Ann Clin Res.

[19] Salonen JT, Nyyssönen K, Korpela H, Tuomilehto J, Seppänen R, Salonen R (1992). High stored iron levels are associated with excess risk of myocardial infarction in eastern Finnish men.. Circulation.

[20] Kunutsor SK, Khan H, Laukkanen JA (2016). Serum magnesium and risk of new onset heart failure in men: the Kuopio Ischemic Heart Disease Study.. Eur J Epidemiol.

[21] Lakka TA, Venäläinen JM, Rauramaa R, Salonen R, Tuomilehto J, Salonen JT (1994). Relation of leisure-time physical activity and cardiorespiratory fitness to the risk of acute myocardial infarction.. N Engl J Med.

[22] Levey AS, Stevens LA, Schmid CH, Zhang YL, Castro AF, Feldman HI, Kusek JW, Eggers P, Van Lente F, Greene T, Coresh J, CKD-EPI (Chronic Kidney Disease Epidemiology Collaboration) (2009). A new equation to estimate glomerular filtration rate.. Ann Intern Med.

[23] Willett W, Willett W (2013). Implications of total energy intake for epidemiologic analyses.. In: Nutritional Epidemiology.

[24] United States Department of Agriculture Agricultural Research Service USDA Food Composition Databases.. https://ndb.nal.usda.gov/ndb/.

[25] Huang Y, Zhou M, Sun H, Wang Y (2011). Branched-chain amino acid metabolism in heart disease: an epiphenomenon or a real culprit?. Cardiovasc Res.

[26] Sun H, Olson KC, Gao C, Prosdocimo DA, Zhou M, Wang Z, Jeyaraj D, Youn JY, Ren S, Liu Y, Rau CD, Shah S, Ilkayeva O, Gui WJ, William NS, Wynn RM, Newgard CB, Cai H, Xiao X, Chuang DT, Schulze PC, Lynch C, Jain MK, Wang Y (2016). Catabolic defect of branched-chain amino acids promotes heart failure.. Circulation.

[27] Tanada Y, Shioi T, Kato T, Kawamoto A, Okuda J, Kimura T (2015). Branched-chain amino acids ameliorate heart failure with cardiac cachexia in rats.. Life Sci.

[28] D’Antona G, Ragni M, Cardile A, Tedesco L, Dossena M, Bruttini F, Caliaro F, Corsetti G, Bottinelli R, Carruba MO, Valerio A, Nisoli E (2010). Branched-chain amino acid supplementation promotes survival and supports cardiac and skeletal muscle mitochondrial biogenesis in middle-aged mice.. Cell Metab.

[29] Wang W, Zhang F, Xia Y, Zhao S, Yan W, Wang H, Lee Y, Li C, Zhang L, Lian K, Gao E, Cheng H, Tao L (2016). Defective branched chain amino acid catabolism contributes to cardiac dysfunction and remodeling following myocardial infarction.. Am J Physiol Heart Circ Physiol.

[30] Tektonidis TG, Åkesson A, Gigante B, Wolk A, Larsson SC (2015). A Mediterranean diet and risk of myocardial infarction, heart failure and stroke: a population-based cohort study.. Atherosclerosis.

[31] Tektonidis TG, Åkesson A, Gigante B, Wolk A, Larsson SC (2016). Adherence to a Mediterranean diet is associated with reduced risk of heart failure in men.. Eur J Heart Fail.

[32] Drouin-Chartier JP, Brassard D, Tessier-Grenier M, Côté JA, Labonté MÈ, Desroches S, Couture P, Lamarche B (2016). Systematic review of the association between dairy product consumption and risk of cardiovascular-related clinical outcomes.. Adv Nutr.

[33] Thorning TK, Raben A, Tholstrup T, Soedamah-Muthu SS, Givens I, Astrup A (2016). Milk and dairy products: good or bad for human health? An assessment of the totality of scientific evidence.. Food Nutr Res.

[34] Gijsbers L, Ding EL, Malik VS, de Goede J, Geleijnse JM, Soedamah-Muthu SS (2016). Consumption of dairy foods and diabetes incidence: a dose-response meta-analysis of observational studies.. Am J Clin Nutr.

[35] Koskinen TT, Virtanen HEK, Voutilainen S, Tuomainen T, Mursu J, Virtanen JK (2017). Fermented vs. non-fermented dairy and risk of coronary heart disease in men: the Kuopio Ischaemic Heart Disease Risk Factor Study [Abstract].. Circulation.

[36] Chartrand D, Da Silva MS, Julien P, Rudkowska I (2017). Influence of amino acids in dairy products on glucose homeostasis: the clinical evidence.. Can J Diabetes.

[37] Pasin G, Comerford KB (2015). Dairy foods and dairy proteins in the management of type 2 diabetes: a systematic review of the clinical evidence.. Adv Nutr.

[38] McGregor RA, Poppitt SD (2013). Milk protein for improved metabolic health: a review of the evidence.. Nutr Metab (Lond).

[39] Schwingshackl L, Schwedhelm C, Hoffmann G, Lampousi AM, Knüppel S, Iqbal K, Bechthold A, Schlesinger S, Boeing H (2017). Food groups and risk of all-cause mortality: a systematic review and meta-analysis of prospective studies.. Am J Clin Nutr.

[40] Nagatomo Y, Tang WH (2015). Intersections between microbiome and heart failure: revisiting the gut hypothesis.. J Card Fail.

[41] Donneyong MM, Hornung CA, Taylor KC, Baumgartner RN, Myers JA, Eaton CB, Gorodeski EZ, Klein L, Martin LW, Shikany JM, Song Y, Li W, Manson JE (2015). Risk of heart failure among postmenopausal women: a secondary analysis of the randomized trial of vitamin D plus calcium of the women’s health initiative.. Circ Heart Fail.

[42] Wang X, Chen H, Ouyang Y, Liu J, Zhao G, Bao W, Yan M (2014). Dietary calcium intake and mortality risk from cardiovascular disease and all causes: a meta-analysis of prospective cohort studies.. BMC Med.

[43] Tankeu AT, Ndip Agbor V, Noubiap JJ (2017). Calcium supplementation and cardiovascular risk: a rising concern.. J Clin Hypertens (Greenwich).

[44] Reid IR, Bolland MJ, Avenell A, Grey A (2011). Cardiovascular effects of calcium supplementation.. Osteoporos Int.

[45] Anderson JJ, Kruszka B, Delaney JA, He K, Burke GL, Alonso A, Bild DE, Budoff M, Michos ED (2016). Calcium intake from diet and supplements and the risk of coronary artery calcification and its progression among older adults: 10-year follow-up of the Multi-Ethnic Study of Atherosclerosis (MESA).. J Am Heart Assoc.

[46] Hou LN, Li F, Zhou Y, Nie SH, Su L, Chen PA, Tan WL, Xu DL (2012). Fish intake and risk of heart failure: a meta-analysis of five prospective cohort studies.. Exp Ther Med.

[47] Mozaffarian D, Bryson CL, Lemaitre RN, Burke GL, Siscovick DS (2005). Fish intake and risk of incident heart failure.. J Am Coll Cardiol.

[48] Belin RJ, Greenland P, Martin L, Oberman A, Tinker L, Robinson J, Larson J, Van Horn L, Lloyd-Jones D (2011). Fish intake and the risk of incident heart failure: the Women’s Health Initiative.. Circ Heart Fail.

[49] Weiss EP (2014). Heart failure risk: effects of red meat, processed red meat, (and enhanced red meat?).. Circ Heart Fail.

[50] Clayton ZS, Fusco E, Kern M (2017). Egg consumption and heart health: a review.. Nutrition.

