# Supplementary material for: Intake of Different Dietary Proteins and Risk of Heart Failure in Men: The Kuopio Ischaemic Heart Disease Risk Factor Study
Source: Circ Heart Fail. 2018 May 29;11(6):e004531. doi: 10.1161/CIRCHEARTFAILURE.117.004531 (PMC6023591; doi:10.1161/CIRCHEARTFAILURE.117.004531)
Supplement: Supplementary file 1 [file hhf-11-e004531-s001.pdf]

SUPPLEMENTAL MATERIAL

Supplemental table 1. Dietary sources of protein among 2441 men from the Kuopio Ischaemic Heart Disease Risk Factor Study

ANIMAL PROTEIN SOURCES								PLANT PROTEIN SOURCES
MEAT				DAIRY		OTHER ANIMAL SOURCES		
**Unprocessed red meat**	**Processed red meat**	**White meat***	**Offal**	**Non-fermented dairy**	**Fermented dairy**	**Fish**	**Eggs**	
Beef and veal	Sausage	Chicken	Liver	Milk	Cheese	Fish	Whole eggs	Grain products
Pork	Bacon	Turkey	Kidney	Cream	Cottage cheese	Shellfish	Eggs in foods and baked goods	Legumes
Mutton and lamb	Store-marinated meats		Heart	Ice cream	Sour milk			Nuts and seeds
Game (elk, reindeer)	Cold cuts		Sweetbread	Milk pudding	Yoghurt			Potatoes
	Canned processed meats		Blood	Powdered milk	Curdled milk			Vegetables
	Meat aspic				Quark			Mushrooms
					Sour cream			Fruits and berries
					Crème fraiche			
					Fromage frais			

*All white meat was unprocessed.

Supplemental table 2. Mean intakes and correlation coefficients[*] between proteins from different dietary sources among 2441 men from the Kuopio Ischaemic Heart Disease Risk Factor Study

	**TOTAL PROTEIN[†]**	**Animal protein**	Total meat[‡]	Red meat	Unpro-cessed red meat	Pro-cessed red meat	Fish	Dairy	Non-fermen-ted dairy	Milk	Fer-mented dairy	Cheese	Egg	**Plant protein**	Grains	Non-grain plant sources
Mean intake, g/d (SD)	93.2 (14.4)	65.2 (15.4)	24.7 (11.4)	22.3 (10.9)	13.2 (8.9)	9.1 (7.2)	8.1 (9.8)	28.8 (11.1)	17.5 (10.0)	16.8 (10.0)	11.3 (9.6)	5.7 (6.8)	3.6 (2.8)	25.8 (6.0)	20.5 (5.7)	5.3 (2.3)
**TOTAL PROTEIN[†]**	1	0.91	0.40	0.34	0.31	0.11	0.34	0.44	0.10	0.11	0.38	0.26	0.04	0.03[§]	-0.01[§]	0.12
**Animal protein**		1	0.47	0.42	0.32	0.21	0.34	0.47	0.16	0.17	0.33	0.22	0.07	-0.34	-0.35	-0.003[§]
Total meat[‡]			1	0.91	0.65	0.51	-0.15	-0.19	-0.21	-0.21	0.002[§]	0.06	-0.04	-0.24	-0.28	0.07
Red meat				1	0.72	0.55	-0.17	-0.18	-0.19	-0.19	-0.01[§]	0.04[§]	-0.03[§]	-0.24	-0.27	0.05
Unprocessed red meat					1	-0.09	-0.13	-0.10	-0.12	-0.12	0.03[§]	0.05	0.01[§]	-0.09	-0.16	0.16
Processed red meat						1	-0.09	-0.13	-0.12	-0.12	-0.03[§]	0.02[§]	-0.06	-0.24	-0.20	-0.11
Fish							1	-0.09	-0.10	-0.09	0.002[§]	-0.02[§]	-0.04	-0.07	-0.08	0.04[§]
Dairy								1	0.56	0.56	0.47	0.27	-0.06	-0.13	-0.10	-0.09
Non-fermented dairy									1	0.99	-0.38	-0.23	0.001[§]	-0.16	-0.11	-0.17
Milk										1	-0.38	-0.23	-0.01[§]	-0.15	-0.09	-0.17
Fermented dairy											1	0.61	-0.03[§]	0.04	0.01[§]	0.08
Cheese												1	0.04	0.05	0.04[§]	0.05
Egg													1	-0.06	-0.05	-0.01[§]
**Plant protein**														1	0.91	0.30
Grains															1	-0.07
Non-grain plant sources																1

[*]Correlation assessed by Spearman correlation coefficient.
[†]Of the mean daily protein intake, 2.2 g was from sources that could not easily be classified as animal or plant protein (for example, dry ready meals and chocolate) and was included into neither animal nor plant protein
[‡]Total meat includes red meat, white meat, and offal.
[§]Non-significant. For all other correlations $P < 0.05$.

Supplemental table 3. Baseline characteristics according to fermented dairy protein intake among 2441 men from the Kuopio Ischaemic Heart Disease Risk Factor Study*

	Fermented dairy protein			
Characteristic	Quartile 1 median intake 1.4 g/d	Quartile 2 median intake 6.9 g/d	Quartile 3 median intake 12.8 g/d	Quartile 4 median intake 22.7 g/d
Subjects, n	610	610	611	610
**Demographic and lifestyle factors**				
Age, y	53.2±5.0	53.1±4.9	52.7±5.3	52.5±5.4†
Education, y	8.0±3.0	8.8±3.4	9.1±3.9	9.0±3.5†
Income, 1000 €	10,1 (8,2)	11,9 (8,7)	12,4 (9,0)	12,6 (10,1)†
Married, %	81.3	87.0	89.0	89.3†
Current smoker, %	41.0	34.3	27.3	25.9†
Regular use of dietary supplements, %	5.2	6.9	7.7	10.3†
Alcohol intake, g/wk	30 (106)	28 (81)	32 (81)	32 (83)
Leisure time physical activity, kcal/d	64 (119)	85 (170)	99 (179)	92 (166)†
Body mass index, kg/m^2	26.8±3.9	26.7±3.4	26.5±3.3	27.0±3.5

**Health and disease status**				
Serum total cholesterol to HDL ratio	4.56 (1.79)	4.53 (1.84)	4.55 (1.88)	4.55 (1.83)
Serum triglycerides, mmol/L	1.07 (0.69)	1.10 (0.73)	1.12 (0.77)	1.10 (0.80)
Serum magnesium, mg/L	19.8±1.5	19.9±1.5	19.9±1.5	19.8±1.6
Systolic blood pressure, mmHg	134±17	134±17	133±17	134±16
Diastolic blood pressure, mmHg	89±11	89±10	88±11	89±10
Estimated glomerular filtration rate, ml/min	87.2±12.0	84.2±12.7	84.8±13.0	85.1±13.0[†]
Family history of CHD, %	46.4	47.2	52.0	46.7
CHD at baseline, %	24.3	21.6	20.6	20.0
CHD during follow-up, %	22.1	22.8	17.8	17.7[†]
Cardiac medication at baseline, %	5.2	2.8	3.6	3.3
Cardiac medication during follow-up, %	62.8	66.7	62.0	62.1
Diabetes at baseline, %	6.2	4.3	3.9	6.7
Diabetes during follow-up, %	20.0	19.7	20.3	21.1
Hypertension at baseline, %	58.4	59.5	57.8	58.2
Hypertension during follow-up, %	26.4	27.0	26.0	26.7
Atrial fibrillation at baseline, %	0.3	0.5	1.6	0.8

Atrial fibrillation during follow-up, %	13.9	16.1	14.4	15.7
Cardiomyopathy at baseline, %	1.3	2.0	1.2	0.8
Cardiomyopathy during follow-up, %	0.8	0.8	1.0	1.1
History of stroke at baseline, %	3.8	2.0	2.1	1.6
Stroke during follow up, %	10.3	10.5	10.5	9.0
Valvular defect during follow-up, %	4.4	5.9	3.8	3.0
Myocarditis during follow-up, %	0.2	0.0	0.3	0.0
Chronic obstructive pulmonary disease at baseline, %	10.0	8.9	8.7	7.9
Chronic obstructive pulmonary disease during follow-up, %	4.6	3.3	2.8	3.3
**Dietary factors**				
Energy, kcal/d	2588±669	2328±600	2351±548	2529±634
Protein, g/d	87.3±14.7	90.4±12.5	93.7±12.5	101.5±14.0[†]
Protein, E%	14.7±2.3	15.4±2.3	15.9±2.4	17.0±2.5[†]
Animal protein, g/d	59.7±16.0	62.2±13.6	65.5±13.5	73.4±15.1[†]
Animal protein, E%	10.0±2.6	10.6±2.5	11.1±2.6	12.3±2.5[†]
Plant protein, g/d	25.4±6.9	25.9±5.6	26.0±5.5	25.9±5.9
Plant protein, E%	4.3±1.1	4.4±1.0	4.4±1.0	4.4±0.9

Fat, E%	39.9±6.4	38.8±5.7	38.0±5.7	38.0±5.6†
SFAs, E%	19.0±4.5	18.0±4.0	17.6±3.8	18.0±3.8†
PUFAs, E%	4.5±1.5	4.6±1.5	4.6±1.4	4.4±1.3
MUFAs, E%	12.0±2.3	11.9±2.2	11.6±2.2	11.4±2.1†
trans Fatty acids, E%	1.1±0.4	1.1±0.4	1.0±0.4	1.1±0.4
Carbohydrates, E%	42.3±6.7	43.1±6.4	43.3±6.4	42.4±6.2
Fiber, g/d	24.5±7.9	24.9±7.0	25.2±6.7	26.0±7.1†
Calcium, mg/d	1117±369	1193±293	1308±299	1576±335†
Fruits, berries and vegetables‡, g/d	194 (183)	222 (201)	241 (211)	258 (192)†
Whole grain products, g/d	154 (99)	141 (88)	143 (85)	161 (90)
Unprocessed red meat, g/d	66 (69)	66 (62)	64 (64)	70 (65)
Processed red meat, g/d	65 (80)	58 (71)	53 (76)	56 (70)†
Fish, g/d	31 (71)	30 (63)	31 (60)	31 (60)
Non-fermented dairy, g/d	704±370	528±301	458±303	401±280†
Fermented dairy g/d	4 (21)	63 (121)	200 (223)	408 (380)†

CHD indicates coronary heart disease; E% percentage of energy intake.

[*]Values are mean ± SD for normally distributed variables, median (interquartile range) for skewed variables and percentages for categorical variables.

[†]*P* for trend across quartiles <0.05.

P-trend was assessed with linear regression (normally distributes variables), Jonckheere Terpstra -test (skewed variables) or with chi-square test (categorical variables).

[‡]Excluding potatoes.

Supplemental table 4. Risk of incident heart failure according to the major protein sources among 2441 men from the Kuopio Ischaemic Heart Disease Risk Factor Study

	Intake quartile					
	1 (n=610)	2 (n=610)	3 (n=611)	4 (n=610)	*P*-trend	Per 100 g increase[*]
Total meat[†]						
Median intake (g/d)	76	126	173	251		
Number of events, incidence rate/1000 PY	96, 7.28	81, 5.95	84, 6.19	73, 5.27		
Model 1	1	0.94 (0.70–1.27)[‡]	1.10 (0.81–1.48)	1.19 (0.86–1.66)	0.22	1.07 (0.92–1.25)
Model 2	1	0.90 (0.67–1.22)	1.09 (0.80–1.47)	1.10 (0.79–1.54)	0.39	1.03 (0.88–1.20)
Model 3	1	0.94 (0.69–1.29)	1.17 (0.83–1.63)	1.23 (0.79–1.90)	0.23	1.07 (0.86–1.34)
Red meat						
Median intake (g/d)	65	113	156	231		
Number of events, incidence rate/1000 PY	81, 6.12	100, 7.42	78, 5.78	75, 5.34		
Model 1	1	1.40 (1.04–1.89)	1.22 (0.89–1.68)	1.41 (1.00–1.97)	0.11	1.10 (0.94–1.29)

Model 2	1	1.44 (1.07–1.94)	1.16 (0.84–1.60)	1.26 (0.90–1.78)	0.37	1.02 (0.87–1.21)
Model 3	1	1.50 (1.10–2.04)	1.22 (0.86–1.73)	1.37 (0.89–2.11)	0.29	1.04 (0.83–1.31)
Processed red meat						
Median intake (g/d)	10	40	76	139		
Number of events, incidence rate/1000 PY	89, 6.64	74, 5.62	93, 6.64	78, 5.71		
Model 1	1	0.90 (0.66–1.22)	1.24 (0.93–1.67)	1.27 (0.92–1.74)	0.05	1.20 (0.99–1.44)
Model 2	1	0.85 (0.62–1.15)	1.05 (0.78–1.41)	0.96 (0.70–1.32)	0.88	1.02 (0.84–1.24)
Model 3	1	0.83 (0.61–1.14)	1.05 (0.77–1.44)	0.94 (0.64–1.38)	0.94	1.02 (0.79–1.31)
Unprocessed red meat						
Median intake (g/d)	21	53	81	132		
Number of events, incidence rate/1000 PY	91, 6.85	90, 6.55	76, 5.67	77, 5.57		
Model 1	1	0.97 (0.72–1.30)	0.95 (0.69–1.29)	1.00 (0.73–1.37)	0.99	0.94 (0.74–1.19)
Model 2	1	1.01 (0.75–1.35)	0.98 (0.72–1.34)	1.14 (0.83–1.57)	0.43	1.02 (0.80–1.29)
Model 3	1	1.00 (0.75–1.35)	0.99 (0.72–1.36)	1.16 (0.84–1.60)	0.39	1.03 (0.81–1.32)
Fish						
Median intake (g/d)	0	18	48	101		
Number of events, incidence rate/1000 PY	91, 6.77	69, 4.96	83, 6.05	91, 6.92		

Model 1	1	0.67 (0.49–0.91)	0.82 (0.61–1.11)	0.99 (0.74–1.32)	0.38	1.08 (0.89–1.33)
Model 2	1	0.60 (0.44–0.83)	0.83 (0.61–1.11)	0.85 (0.63–1.14)	0.88	0.98 (0.80–1.20)
Model 3	1	0.62 (0.45–0.85)	0.84 (0.62–1.13)	0.89 (0.66–1.21)	0.64	1.01 (0.82–1.25)
Egg						
Median intake (g/d)	8	20	34	59		
Number of events, incidence rate/1000 PY	88, 7.15	85, 6.14	85, 6.03	76, 5.43		
Model 1	1	0.82 (0.61–1.11)	0.80 (0.59–1.09)	0.75 (0.54–1.03)	0.12	0.94 (0.74–1.20)*
Model 2	1	0.96 (0.71–1.30)	0.96 (0.71–1.31)	0.89 (0.64–1.22)	0.47	1.01 (0.80–1.28)*
Model 3	1	0.99 (0.73–1.35)	0.99 (0.73–1.35)	0.92 (0.66–1.27)	0.59	1.04 (0.82–1.32)*
Dairy products						
Median intake (g/d)	286	572	800	1122		
Number of events, incidence rate/1000 PY	78, 5.66	73, 5.36	88, 6.53	95, 7.11		
Model 1	1	0.98 (0.71–1.36)	1.20 (0.87–1.65)	1.62 (1.14–2.29)	0.004	1.06 (1.02–1.10)
Model 2	1	0.89 (0.64–1.23)	1.03 (0.75–1.42)	1.36 (0.95–1.94)	0.07	1.04 (1.00–1.08)
Model 3	1	0.94 (0.68–1.31)	1.09 (0.78–1.52)	1.48 (1.02–2.16)	0.03	1.05 (1.01–1.09)
Non-fermented dairy products						
Median intake (g/d)	156	371	585	909		

Number of events, incidence rate/1000 PY	84, 6.12	74, 5.42	99, 7.26	77, 5.83		
Model 1	1	0.86 (0.63–1.17)	1.16 (0.86–1.56)	1.12 (0.79–1.57)	0.24	1.03 (0.99–1.07)
Model 2	1	0.82 (0.59–1.12)	0.96 (0.71–1.30)	0.93 (0.65–1.31)	0.94	1.00 (0.96–1.04)
Model 3	1	0.84 (0.61–1.16)	1.00 (0.73–1.36)	0.95 (0.66–1.37)	0.98	1.01 (0.96–1.05)
Milk						
Median intake (g/d)	140	349	564	876		
Number of events, incidence rate/1000 PY	85, 6.17	71, 5.19	105, 7.71	73, 5.55		
Model 1	1	0.81 (0.59–1.11)	1.23 (0.92–1.65)	1.07 (0.76–1.51)	0.24	1.03 (0.99–1.07)
Model 2	1	0.78 (0.57–1.07)	1.02 (0.76–1.37)	0.87 (0.61–1.24)	0.84	1.00 (0.96–1.04)
Model 3	1	0.80 (0.58–1.11)	1.07 (0.79–1.45)	0.89 (0.62–1.28)	0.94	1.01 (0.97–1.05)
Fermented dairy products						
Median intake (g/d)	3	55	179	435		
Number of events, incidence rate/1000 PY	76, 5.89	75, 5.36	73, 5.19	110, 8.29		
Model 1	1	0.96 (0.69–1.32)	0.84 (0.61–1.16)	1.44 (1.07–1.93)	0.003	1.06 (1.01–1.11)
Model 2	1	1.19 (0.86–1.64)	1.00 (0.72–1.38)	1.62 (1.20–2.19)	0.001	1.06 (1.01–1.11)
Model 3	1	1.22 (0.88–1.68)	1.02 (0.74–1.42)	1.69 (1.25–2.29)	0.001	1.06 (1.01–1.12)
Cheese						

Median intake (g/d)	0	9	24	50		
Number of events, incidence rate/1000 PY	102, 7.39	91, 6.53	77, 5.82	64, 4.82		
Model 1	1	0.82 (0.62–1.09)	0.78 (0.58–1.05)	0.81 (0.59–1.11)	0.24	0.85 (0.66–1.10)*
Model 2	1	1.02 (0.76–1.36)	0.99 (0.73–1.35)	1.20 (0.86–1.68)	0.31	1.14 (0.88–1.47)*
Model 3	1	1.05 (0.79–1.41)	1.01 (0.74–1.37)	1.22 (0.87–1.70)	0.30	1.13 (0.88–1.45)*
Other fermented dairy§						
Median intake (g/d)	0	50	185	446		
Number of events, incidence rate/1000 PY‖	158, 8.82	95, 7.57	92, 7.57	124, 10.66		
Model 1	1	0.85 (0.62–1.15)	0.83 (0.61–1.13)	1.40 (1.06–1.85)	0.004	1.07 (1.02–1.12)
Model 2	1	0.96 (0.71–1.32)	0.91 (0.66–1.24)	1.45 (1.09–1.92)	0.006	1.06 (1.01–1.11)
Model 3	1	1.00 (0.73–1.37)	0.93 (0.68–1.27)	1.50 (1.13–2.00)	0.003	1.06 (1.01–1.11)
Major plant protein sources#						
Median intake (g/d)	163	221	278	367		
Number of events, incidence rate/1000 PY	91, 7.14	89, 6.61	77, 5.49	77, 5.50		
Model 1	1	0.97 (0.72–1.31)	0.81 (0.58–1.12)	0.94 (0.65–1.36)	0.59	0.94 (0.80–1.11)
Model 2	1	1.10 (0.81–1.49)	0.93 (0.66–1.30)	1.11 (0.76–1.63)	0.76	0.99 (0.84–1.16)
Model 3	1	1.09 (0.79–1.51)	0.92 (0.64–1.34)	1.06 (0.66–1.71)	0.98	0.92 (0.73–1.16)

PY indicates person-years.

*For egg and cheese intakes the HRs (95% CIs) in the continuous models are presented per 50 g increase in intake, because of the low average intake compared to other food groups.

†Total meat includes red meat, white meat and offal.

‡Values are HRs (95% CIs) derived from Cox proportional hazards regression models.

§Other fermented dairy includes sour milk, yoghurt, curdled milk, quark, sour cream and crème fraiche.

||Number of subjects in the quartiles one to four: 829 (zero intake), 539, 537, and 536, respectively.

#Major plant protein sources include grain products, legumes, nuts, and seeds.

Model 1 adjusted for age (y), examination year, and energy intake (kcal/d).

Model 2 adjusted for Model 1 and education (y), income (euros/y), pack-years of smoking (packs smoked per day × years smoked), alcohol intake (g/week), leisure-time physical activity (kcal/d), body mass index (kg/m^2), family history of coronary heart disease (yes/no), diseases (coronary heart diseases or use of cardiac medications, diabetes or hypertension) at the baseline and during the follow-up.

Model 3 adjusted for Model 2 and intakes of saturated (g/d), monounsaturated (g/d), polyunsaturated (g/d), and trans fatty acids (g/d) and fiber (g/d).

Supplemental table 5. Risk of incident heart failure according to the dietary calcium intake among 2441 men from the Kuopio Ischaemic Heart Disease Risk Factor Study

	Intake quartile					
	1 (n=610)	2 (n=610)	3 (n=611)	4 (n=610)	*P*-trend	Per 100 mg increase
Calcium intake						
Median intake (mg/d)	775	1116	1409	1849		
Number of events, incidence rate/1000 PY*	71, 5.17	87, 6.35	73, 5.42	103, 7.72		
Model 1	1	1.13 (0.83–1.55)*	0.95 (0.69–1.32)	1.47 (1.09–2.00)	0.02	1.04 (1.01–1.07)
Model 2	1	1.09 (0.79–1.51)	0.91 (0.65–1.27)	1.39 (1.01–1.91)	0.07	1.05 (1.01–1.08)

PY indicates person-years.

*Values are HRs (95% CIs) derived from Cox proportional hazards regression models.

Model 1 adjusted for age (y), examination year, and energy intake (kcal/d).

Model 2 adjusted for Model 1 and education (y), income (euros/y), pack-years of smoking (packs smoked per day × years smoked), alcohol intake (g/week), leisure-time physical activity (kcal/d), body mass index (kg/m^2), family history of coronary heart disease (yes/no), diseases (coronary heart diseases or use of cardiac medications, diabetes or hypertension) at the baseline and during the follow-up, and intakes of saturated (g/d), monounsaturated (g/d), polyunsaturated (g/d), and trans fatty acids (g/d) and fiber (g/d).

Supplemental Table 6. Risk of composite outcome of heart failure or cardiovascular disease death according to protein intake among 1640 men free of cardiovascular disease from the Kuopio Ischaemic Heart Disease Risk Factor Study

	Intake quartile					
	1 (n=410)	2 (n=410)	3 (n=410)	4 (n=410)	*P*-trend	Per 5 g increase
**TOTAL PROTEIN**						
Median intake (g/d)	78.3	88.3	96.8	109.7		
Number of events, incidence rate/1000 PY	104, 11.04	91, 9.59	85, 8.91	99, 10.56		
Model 1	1	0.90 (0.68–1.20)*	0.88 (0.66–1.78)	1.13 (0.85–1.49)	0.42	1.02 (0.96–1.08)
Model 2	1	0.97 (0.73–1.30)	1.03 (0.77–1.39)	1.26 (0.93–1.69)	0.12	1.03 (0.98–1.09)
**Animal protein**						
Median intake (g/d)	49.0	60.3	69.0	81.9		
Number of events, incidence rate/1000 PY	100, 10.58	87, 8.92	93, 10.00	99, 10.62		
Model 1	1	0.86 (0.64–1.14)	1.04 (0.78–1.39)	1.20 (0.90–1.58)	0.12	1.02 (0.97–1.07)
Model 2	1	0.83 (0.62–1.12)	1.06 (0.78–1.43)	1.20 (0.87–1.65)	0.13	1.03 (0.98–1.09)
Protein from total meat†						
Median intake (g/d)	12.6	20.5	27.5	38.2		
Number of events, incidence rate/1000 PY	105, 11.29	99, 10.44	93, 9.73	82, 8.65		

Model 1	1	0.99 (0.75–1.31)	0.99 (0.75–1.31)	1.01 (0.76–1.36)	0.94	1.00 (0.93–1.08)
Model 2	1	1.00 (0.74–1.34)	1.06 (0.77–1.45)	1.12 (0.77–1.63)	0.50	1.01 (0.92–1.11)
Protein from red meat						
Median intake (g/d)	10.5	18.2	25.0	34.6		
Number of events, incidence rate/1000 PY	104, 11.25	91, 9.46	99, 10.52	85, 8.89		
Model 1	1	0.87 (0.66–1.16)	1.10 (0.84–1.45)	0.98 (0.74–1.32)	0.75	1.00 (0.93–1.07)
Model 2	1	0.96 (0.71–1.30)	1.18 (0.86–1.62)	1.09 (0.75–1.58)	0.46	1.00 (0.90–1.10)
Protein from processed red meat						
Median intake (g/d)	1.7	5.9	10.2	17.0		
Number of events, incidence rate/1000 PY	79, 8.44	103, 11.04	104, 10.92	93, 9.68		
Model 1	1	1.38 (1.03–1.85)	1.46 (1.09–1.96)	1.41 (1.04–1.90)	0.04	1.08 (0.97–1.20)
Model 2	1	1.40 (1.04–1.90)	1.34 (0.97–1.84)	1.33 (0.91–1.95)	0.24	1.02 (0.87–1.18)
Protein from unprocessed red meat						
Median intake (g/d)	4.0	9.6	14.8	23.8		
Number of events, incidence rate/1000 PY	100, 10.63	113, 11.96	91, 9.81	75, 7.74		
Model 1	1	1.10 (0.84–1.44)	0.94 (0.71–1.25)	0.80 (0.59–1.08)	0.07	0.94 (0.86–1.03)
Model 2	1	1.09 (0.83–1.45)	0.99 (0.73–1.33)	0.95 (0.68–1.34)	0.63	0.99 (0.89–1.10)

Protein from fish						
Median intake (g/d)	0	3.0	8.1	17.5		
Number of events, incidence rate/1000 PY	95, 9.95	97, 10.10	87, 9.14	100, 10.91		
Model 1	1	0.96 (0.72–1.28)	0.86 (0.64–1.16)	1.02 (0.77–1.35)	0.85	1.01 (0.93–1.09)
Model 2	1	0.95 (0.70–1.27)	0.88 (0.65–1.19)	0.93 (0.69–1.26)	0.68	0.99 (0.90–1.09)
Protein from egg						
Median intake (g/d)	1.1	2.4	3.9	6.5		
Number of events, incidence rate/1000 PY	113, 12.81	87, 8.83	87, 9.05	92, 9.64		
Model 1	1	0.62 (0.47–0.83)	0.62 (0.47–0.83)	0.70 (0.53–0.92)	0.05	0.79 (0.57–1.08)
Model 2	1	0.73 (0.55–0.98)	0.74 (0.55–0.99)	0.81 (0.61–1.09)	0.31	0.86 (0.63–1.19)
Protein from dairy						
Median intake (g/d)	17.0	25.1	31.6	40.4		
Number of events, incidence rate/1000 PY	87, 9.07	95, 10.06	86, 8.97	111, 12.06		
Model 1	1	1.05 (0.79–1.41)	0.92 (0.68–1.24)	1.35 (1.02–1.78)	0.07	1.05 (0.98–1.13)
Model 2	1	1.07 (0.80–1.44)	1.00 (0.73–1.38)	1.40 (1.02–1.92)	0.05	1.08 (0.99–1.17)
Protein from non-fermented dairy						
Median intake (g/d)	6.2	13.1	19.6	29.2		

Number of events, incidence rate/1000 PY	79, 8.04	91, 9.74	94, 9.81	115, 12.67		
Model 1	1	1.14 (0.84–1.54)	1.10 (0.81–1.48)	1.47 (1.11–1.97)	0.009	1.05 (0.97–1.13)
Model 2	1	1.25 (0.91–1.71)	1.06 (0.77–1.47)	1.56 (1.10–2.21)	0.005	1.07 (0.97–1.18)
Protein from milk						
Median intake (g/d)	5.6	12.3	18.9	28.6		
Number of events, incidence rate/1000 PY	72, 7.28	97, 10.43	99, 10.34	111, 12.25		
Model 1	1	1.36 (1.00–1.85)	1.31 (0.96–1.78)	1.59 (1.18–2.14)	0.005	1.05 (0.97–1.14)
Model 2	1	1.45 (1.05–1.99)	1.25 (0.90–1.74)	1.60 (1.11–2.30)	0.006	1.06 (0.96–1.18)
Protein from fermented dairy						
Median intake (g/d)	1.3	7.2	13.0	22.7		
Number of events, incidence rate/1000 PY	104, 11.36	83, 8.72	94, 9.70	98, 10.36		
Model 1	1	0.74 (0.55–0.98)	0.85 (0.64–1.13)	0.97 (0.74–1.28)	0.77	1.02 (0.94–1.10)
Model 2	1	0.86 (0.64–1.16)	1.24 (0.92–1.68)	1.44 (1.05–1.99)	0.006	1.09 (0.99–1.20)
Protein from cheese						
Median intake (g/d)	-0.1	2.3	6.2	13.8		
Number of events, incidence rate/1000 PY	116, 12.83	102, 10.88	81, 8.34	80, 8.24		
Model 1	1	0.83 (0.63–1.09)	0.65 (0.49–0.87)	0.77 (0.57–1.03)	0.08	0.86 (0.75–0.99)

Model 2	1	0.93 (0.70–1.22)	0.84 (0.63–1.13)	1.20 (0.87–1.65)	0.26	1.02 (0.88–1.18)
Protein from other fermented dairy‡						
Median intake (g/d)	-0.5	1.2	5.1	13.7		
Number of events, incidence rate/1000 PY	89, 9.40	89, 9.47	95, 9.81	106, 11.42		
Model 1	1	0.90 (0.65–1.24)	0.92 (0.67–1.24)	1.05 (0.79–1.41)	0.41	1.12 (1.02–1.23)
Model 2	1	0.97 (0.70, 1.34)	1.10 (0.81, 1.50)	1.23 (0.90, 1.69)	0.11	1.14 (1.02–1.28)
**Plant protein**						
Median intake (g/d)	19.8	23.9	27.4	32.4		
Number of events, incidence rate/1000 PY	106, 11.70	85, 8.96	97, 10.14	91, 9.36		
Model 1	1	0.70 (0.53–0.94)	0.77 (0.58–1.02)	0.72 (0.54–0.95)	0.05	0.96 (0.83–1.10)
Model 2	1	0.83 (0.61–1.15)	1.11 (0.78–1.59)	1.13 (0.72–1.77)	0.36	1.11 (0.85–1.43)
Protein from grain products						
Median intake (g/d)	14.7	18.6	21.9	26.9		
Number of events, incidence rate/1000 PY	103, 11.29	89, 9.35	95, 9.96	92, 9.54		
Model 1	1	0.77 (0.58–1.03)	0.78 (0.59–1.03)	0.78 (0.58–1.03)	0.11	0.97 (0.84–1.12)
Model 2	1	0.89 (0.65–1.22)	0.98 (0.69–1.40)	1.08 (0.69–1.68)	0.63	1.10 (0.85–1.43)
Protein from non-grain plant protein sources						

Median intake (g/d)	3.0	4.4	5.7	7.8		
Number of events, incidence rate/1000 PY	100, 10.75	105, 11.11	80, 8.35	94, 9.89		
Model 1	1	0.99 (0.76–1.31)	0.75 (0.56–1.01)	0.93 (0.70–1.23)	0.37	0.97 (0.84–1.12)
Model 2	1	1.31 (0.98–1.76)	0.95 (0.69–1.31)	1.31 (0.92–1.85)	0.32	1.10 (0.85–1.43)

PY indicates person-years.

*Values are HRs (95% CIs) derived from Cox proportional hazards regression models.

†Total meat includes red meat, white meat and offal.

‡Protein from other fermented dairy includes all the fermented dairy products excluding cheese, i.e. sour milk, yoghurt, curdled milk, quark, sour cream and crème fraiche.

Model 1 adjusted for age (y), examination year, and energy intake (kcal/d).

Model 2 adjusted for Model 1 and education (y), income (euros/y), pack-years of smoking (packs smoked per day × years smoked), alcohol intake (g/week), leisure-time physical activity (kcal/d), body mass index (kg/m^2), family history of coronary heart disease (yes/no), diseases (diabetes or hypertension or use of cardiac medications) at the baseline and during the follow-up, and intakes of saturated (g/d), monounsaturated (g/d), polyunsaturated (g/d), and trans fatty acids (g/d) and fiber (g/d). Model 2 was also mutually adjusted for other proteins.

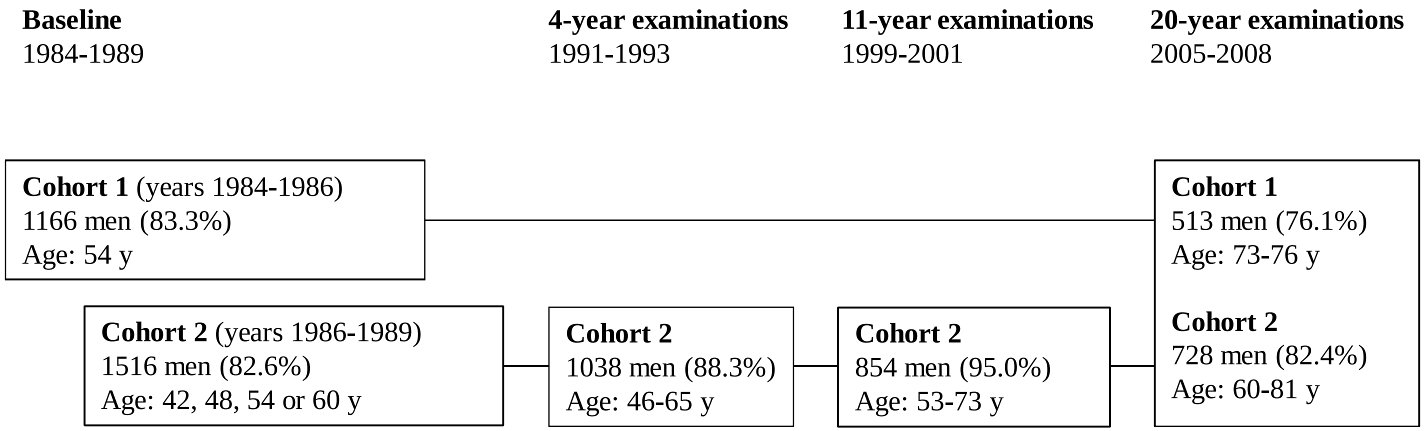

Supplemental Figure 1. Timeline of the Kuopio Ischemic Heart Disease Risk Factor Study.
The percentages in parentheses indicate the proportion of eligible participants that participated in the study visits.

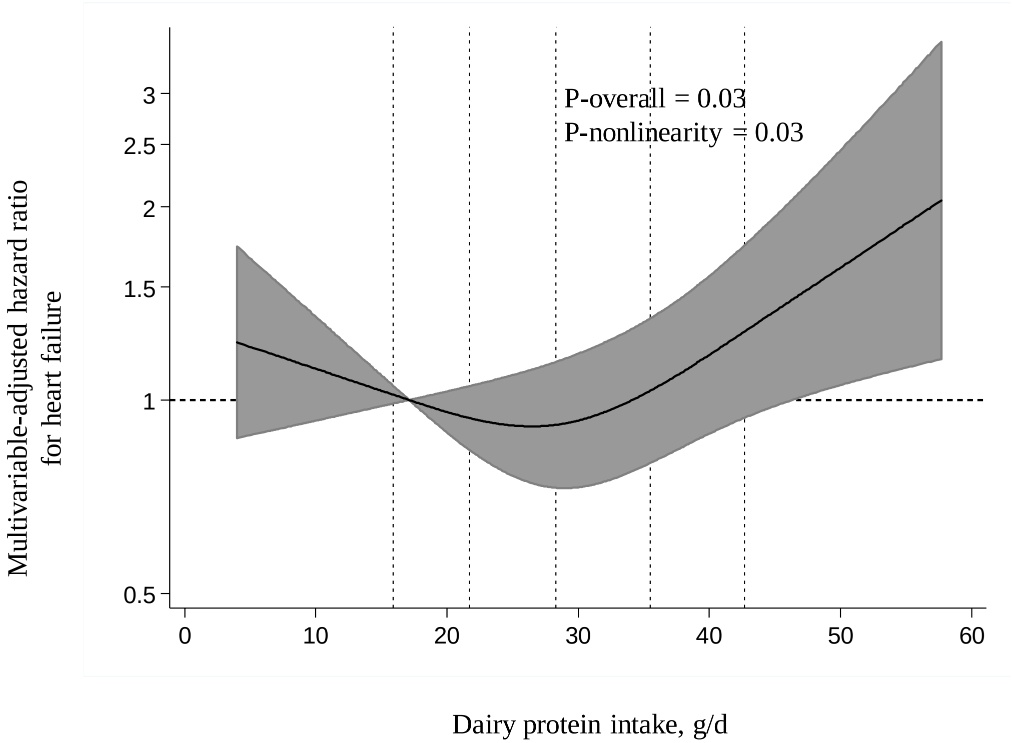

Supplemental Figure 2. Multivariable-adjusted hazard ratios of dairy protein intake with risk of heart failure in 2441 men, evaluated by restricted cubic splines from Cox proportional hazards models. The model is adjusted for age (y), examination year energy intake (kcal/d), education (y), income (euros/y), pack-years of smoking (packs smoked per day × years smoked), alcohol intake (g/week), leisure-time physical activity (kcal/d), body mass index (kg/m^2), family history of coronary heart disease (yes/no), diseases (coronary heart diseases or use of cardiac medications, diabetes or hypertension) at the baseline and during the follow-up, and intakes of saturated (g/d), monounsaturated (g/d), polyunsaturated (g/d), and trans fatty acids (g/d) and fiber (g/d). Model was also mutually adjusted for other proteins (meat, fish, egg and plant protein). The solid lines represent the central risk estimates and the shades are the 95% confidence interval, relative to the reference level (12.5th percentile). The dotted vertical lines correspond to 10th, 25th, 50th, 75th and 90th percentile of the dairy protein intake.
